# Mapping Method of Human Arm Motion Based on Surface Electromyography Signals

**DOI:** 10.3390/s24092827

**Published:** 2024-04-29

**Authors:** Yuanyuan Zheng, Gang Zheng, Hanqi Zhang, Bochen Zhao, Peng Sun

**Affiliations:** 1 School of Mechanical and Energy Engineering, Zhejiang University of Science and Technology, Hangzhou 310023, China; 2 Key Laboratory of Special Purpose Equipment and Advanced Processing Technology, Ministry of Education and Zhejiang Province, Zhejiang University of Technology, Hangzhou 310023, China; 3 College of Mechanical Engineering, Zhejiang University of Technology, Hangzhou 310023, China

**Keywords:** sEMG, gesture recognition, human arm motion mapping, deep learning

## Abstract

This paper investigates a method for precise mapping of human arm movements using sEMG signals. A multi-channel approach captures the sEMG signals, which, combined with the accurately calculated joint angles from an Inertial Measurement Unit, allows for action recognition and mapping through deep learning algorithms. Firstly, signal acquisition and processing were carried out, which involved acquiring data from various movements (hand gestures, single-degree-of-freedom joint movements, and continuous joint actions) and sensor placement. Then, interference signals were filtered out through filters, and the signals were preprocessed using normalization and moving averages to obtain sEMG signals with obvious features. Additionally, this paper constructs a hybrid network model, combining Convolutional Neural Networks and Artificial Neural Networks, and employs a multi-feature fusion algorithm to enhance the accuracy of gesture recognition. Furthermore, a nonlinear fitting between sEMG signals and joint angles was established based on a backpropagation neural network, incorporating momentum term and adaptive learning rate adjustments. Finally, based on the gesture recognition and joint angle prediction model, prosthetic arm control experiments were conducted, achieving highly accurate arm movement prediction and execution. This paper not only validates the potential application of sEMG signals in the precise control of robotic arms but also lays a solid foundation for the development of more intuitive and responsive prostheses and assistive devices.

## 1. Introduction

As society progresses, the demand for human–computer interaction control becomes more and more diversified, complex, and high-precision. Assistive exoskeletons, artificial neural prostheses, and rehabilitative orthotic training devices, among other rehabilitation equipment, are all realized through human–computer interaction control technology [1,2]. Biometric signals contain rich information on limb movements, by analyzing these signals for human movement intention recognition, a more real-time and coordinated human–computer interaction control can be achieved [3]. Specifically, human–computer interaction methods based on sEMG signals are advanced and unaffected by limb integrity, making them suitable not only for general users but also for meeting the human–computer interaction needs of people with disabilities [4].

sEMG signals were first applied in gesture recognition. Zhang, W.L. et al. [5] analyzed time domain features, selecting five feature values as input, and employed an artificial neural network to recognize electromyogram data collected from the upper limb. This method successfully classified eight different hand gestures, achieving a final recognition rate of 89.31%. Zhao, S.H. et al. processed eight-channel sEMG signals using a moving average and segmented the processed results to extract their time-domain features, then recognized four types of hand gestures by using a dynamic time warping algorithm, with a successful recognition rate of up to 94.7% [6]. Kang, S.Y. et al. collected sEMG signals from the arm and improved the online recognition accuracy of hand gestures using deep learning methods, achieving a recognition rate of 90% with network data testing [7]. As the number of recognized hand gestures increases, the recognition accuracy tends to decrease, mainly due to the selection of rather limited features. Therefore, expanding the range of feature selection and using deep learning to replace traditional classification methods, such as Support Vector Machines (SVM) or neural networks, can significantly enhance the recognition rate [8,9]. These studies highlight the potential of deep learning in improving recognition accuracy and the need to expand feature selection to accommodate complex gesture recognition.

Although sEMG signals can classify and recognize discrete motion patterns such as clenched fists and extended palms, they are unable to continuously estimate human motion intention. Continuous estimation of the amount of human joint motion is key to achieving flexible and coordinated movement between rehabilitation motion assistance devices and the human body. Jiang et al. [10] established a Hill-type muscle model [11,12] and a joint geometric parameter model, utilizing sEMG signals to estimate joint torque, which enables continuous estimation of single-joint movement. However, human skeletal muscle parameters are difficult to measure, and model complexity limits their application to situations with few degrees of freedom. Liu, Q. et al. [13] used principal component analysis to extract the principal components of multi-channel sEMG signals. They established a high-order polynomial model from principal components to upper limb joint angles, achieving estimations of joint angles. Still, this approach cannot explain the influence of each sEMG channel on joint angle estimations [14,15]. For patients with limb movement disorders, as well as factors like actual motion interference and sweating, it becomes impossible to monitor sEMG signals directly related to joint movement, and usually, only the sEMG signals from remaining normal functioning muscles can be utilized [16,17].

Based on the aforementioned considerations, this paper focuses on the research of the sEMG signal-joint angle model and processes feature extraction of sEMG signals. Utilizing a BP neural network, we established a nonlinear relationship between sEMG signals and joint angles. Next, we predicted the individual degree of freedom motions and combined motions for the shoulder and elbow joints. Finally, we conducted a humanoid robotic arm control experiment, which successfully achieved the prediction and execution of arm movements.

## 2. Signal Acquisition Strategy

### 2.1. The Placement of sEMG Acquisition Device

**Figure 1:**
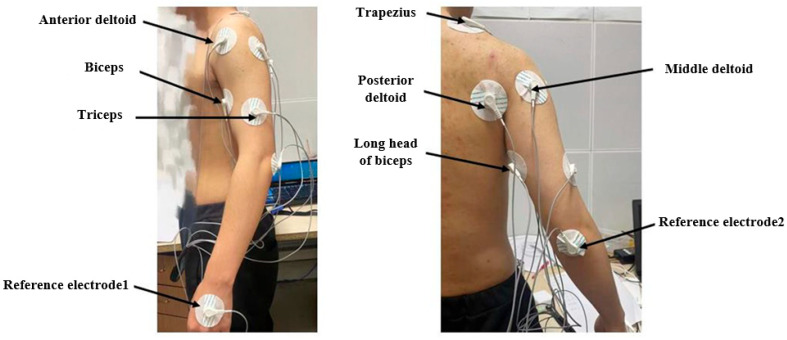
Distribution of sEMG signal acquisition points.

By analyzing the physiological structure and movement patterns of the human arm, seven muscles that are most directly related to the movements of the shoulder joint, wrist joint, and forearm were selected as the subjects for sEMG signals collection. These muscles were the trapezius, long head muscle, the biceps brachii, the triceps brachii, and the anterior, middle, and posterior parts of the deltoid muscle. Furthermore, the back of the hand and the elbow joint, two body parts with no prominent electromyographic signals, were chosen as the sites for the reference electrodes. The arm electromyography signal acquisition device adopts the BClduino electromyography device [18], accompanied by the EMGduino electromyography amplifier lead wires. The electrode patches are affixed to the middle part of each muscle, and the patch position is shown in Figure 1.

On the other hand, as for manual actions, only a few predetermined hand gestures are recognized, such as clenching fingers and flipping the palm up and down. According to the plan, these hand gestures are designed to coordinate the grasping function of a humanoid robotic arm. This is mainly due to the wide distribution of muscle groups that control the hand (mainly the fingers), making them difficult to collect [19,20,21]. Therefore, it is challenging to achieve mapping of fine hand movements based on surface sEMG signals.

**Figure 2:**
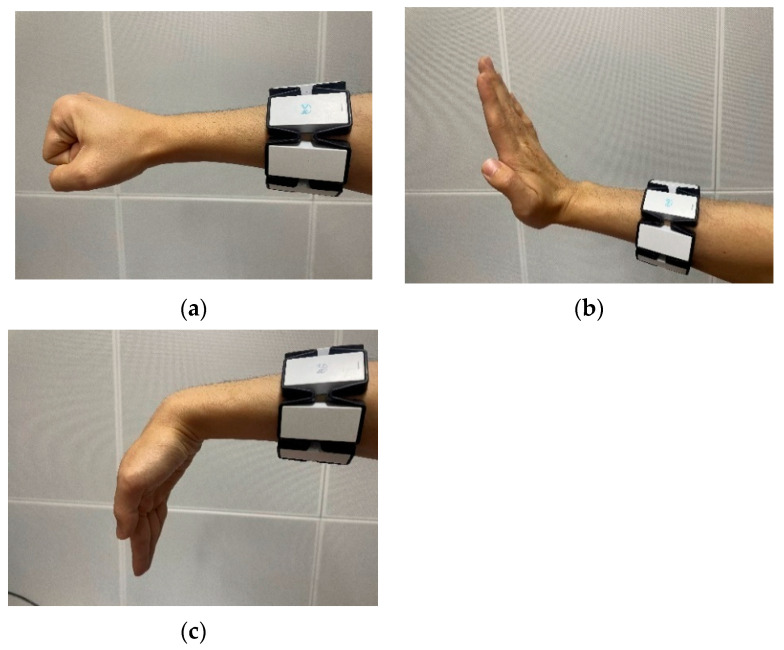
The MYO bracelet wearing position and schematic diagram of gesture action: (**a**) Clenching the palm. (**b**) Flipping up the palm. (**c**) Flipping down the palm.

The gesture acquisition device used is the MYO armband, which is worn just above the wrist, near the middle of the forearm. The muscle activity in this area can control most hand gestures. The wearing position of the MYO armband is shown in Figure 2.

### 2.2. The Placement of Joint Angle Sensors

**Figure 3:**
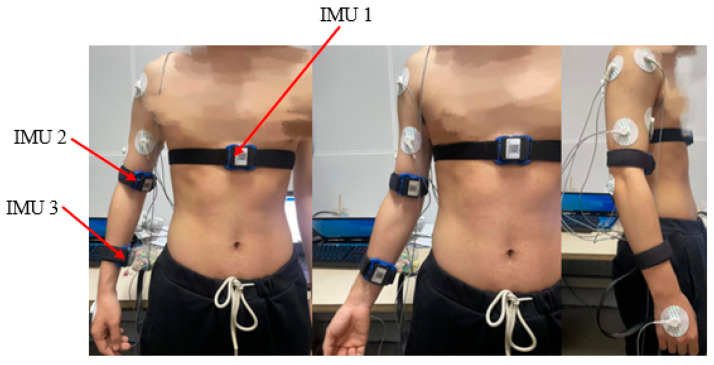
IMU wearing mode.

Using IMU sensors to calculate the motion angles of each joint of the human arm is analogous to solving the inverse kinematics solution of a series-parallel hybrid robotic arm [22,23,24]. We employed a total of three IMU sensors (designated as IMU-Base, IMU-Shoulder, and IMU-Elbow). The IMU-Base is worn at the chest position where there is no limb movement, serving to calibrate the zero positions for the IMU-Shoulder and IMU-Elbow sensors. The IMU-Shoulder is worn on the upper arm to calculate the angular changes of the three degrees of freedom at the shoulder joint. The IMU-Elbow is worn on the forearm to measure the angular changes of the two degrees of freedom at the elbow joint with the forearm. The wearing positions of the IMU sensors are illustrated in Figure 3.

### 2.3. The Setting of Arm Movements

#### 2.3.1. Gesture Actions

**Figure 4:**
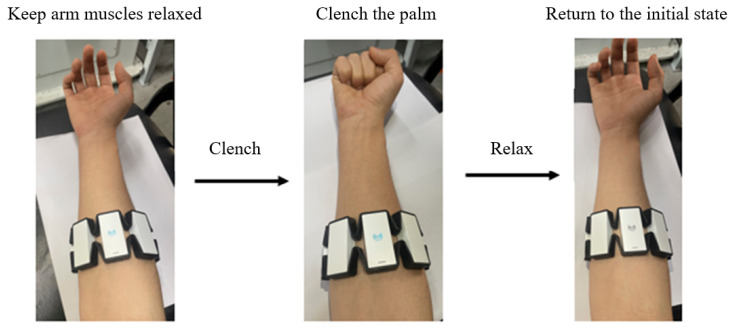
Experimental action flow chart.

The first gesture is clenching the palm into a fist, which corresponds to the command for the robotic claw to close in order to grasp an object. Conversely, relaxing the fist corresponds to the command for the robotic claw to open, as shown in Figure 2a. Secondly, flipping the palm upward has been designated as the emergency stop gesture, considering that there may be dangerous situations during the movement of the mechanical arm, to ensure the safety of the robot arm operation, as shown in Figure 2b. Moreover, after ensuring the elimination of danger and the safety of the operational area, flipping the palm downward has been set as the command to restart the robotic arm, allowing it to resume movement, as displayed in Figure 2c. The collected signals consist of two parts: one is the training set used for algorithm training, and the other is the test set used to assess the algorithm’s recognition performance. The flow of a single group action is presented in Figure 4 (taking the fist-clenching action as an example).

The specific data collection experimental steps are as follows:

1. The participant maintains a comfortable sitting position, with the left arm relaxed and hanging by the side of the body and the right forearm bent and placed on the table in a relaxed state. The right hand performs a gesture and holds it for 3 s. After completing a full gesture, the hand returns to the original state and relaxes for 5 s.
2. Each set of movements is performed five times consecutively. Each action should strive to exert the same force as the previous action. After completing a set of movements, stop collecting the EMG signal, rest for 2–3 min, and once ready, start the next type of movement. Continue collecting data sequentially until the entire dataset has been gathered.
3. The data collection process for the test set and training set is the same, and the data for the training set and test set are collected once in the same order and manner.

#### 2.3.2. Single-Degree-of-Freedom Joint Movement

Based on the range of human arm joint movement, we established the range of motion for a single-degree-of-freedom joint in our data collection experiment. Each range of motion for a single-degree-of-freedom is divided into five groups, as shown in Table 1.

**Figure 5:**
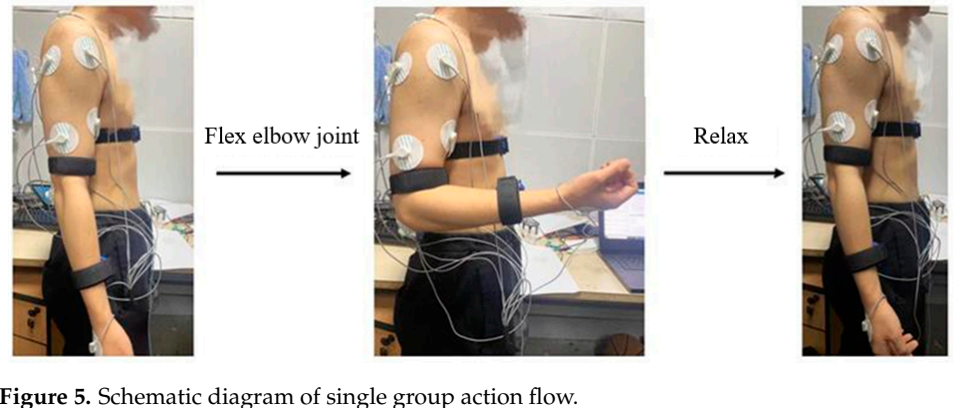
Schematic diagram of single group action flow.

Before the formal start of the experiment, the participant should be acquainted with the entire experimental procedure and safety precautions. This means that the participants should practice the experimental movements without wearing sEMG signal acquisition devices and IMU sensors to ensure the overall success of the experiment. Taking the collection of a single set of motion signals for elbow joint flexing at 90° as an example, as shown in Figure 5.

The specific collection experimental steps are as follows:

1. The participant maintains a standing posture, arms relaxed and hanging vertically at the sides of the body. Starting from the elbow joint as the pivot point, the arms are steadily extended outwards away from the body, ensuring minimal additional movements so that the angle between the forearm and upper arm is approximately 90°. This position is held for 3 s before returning the arms to the initial position (relaxing for 5 s). This step is repeated consecutively five times, with each repetition striving to exert a similar force as the previous one.
2. For the same single-degree-of-freedom motion of a joint, perform other groups of actions in the order described above. After completing a complete joint single-degree-of-freedom movement, stop the collection of sEMG signals and rest for 2–3 min. Ensure good condition before starting the next single-degree-of-freedom movement of the joint. Collect in sequence until all single-degree-of-freedom movement data of all joints are collected.
3. The data collection process for the test set and training set is the same, and the data for the training set and test set are collected once in the same order and manner.

#### 2.3.3. Coordinated Motion of Multiple Joints

**Figure 6:**
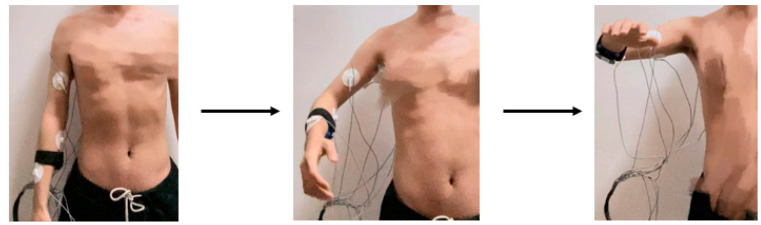
Decomposition example of abduction and raising of the arm.

**Figure 7:**
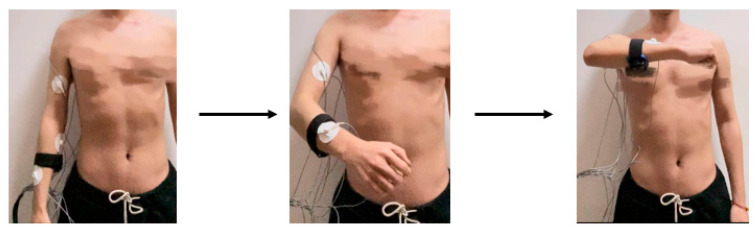
Decomposition example of cross arm with palm clenched.

Both of the above experiments are focused on collecting data from individual joint movements with a single degree of freedom. However, in actual human arm movements, there is a combination of motion of multiple joints. To verify whether there are differences in sEMG signals between joint single-degree-of-freedom motions and multi-joint collaborative motions, two combination movements of abduction and raising of the arm and cross arm with the palm clenched were designed. The outward hand lift movement includes shoulder joint abduction/adduction, elbow joint flexion/extension, and forearm rotation, as shown in Figure 6. The cross arm with palm clenched movement involves shoulder flexion/extension, elbow flexion/extension, forearm rotation, and hand clenching movements, as illustrated in Figure 7. The specific experimental procedures and considerations are consistent with the joint single-degree-of-freedom motion acquisition experiment.

## 3. Gesture Action Recognition

### 3.1. sEMG Signal Processing

By amplifying, filtering [25], and other operations to remove interference from other noises in the sEMG signal, we then extracted the characteristics from the filtered sEMG signal. We used normalization methods [26] to eliminate individual differences between sEMG signals. At the same time, we used the Moving Average Method (MAF) to reduce the impact of short-term random fluctuations on the overall trend of the sEMG signal. Finally, we calculated the mean and variance of the signal after applying the MAF to obtain sEMG signals with distinct features, laying the foundation for subsequent gesture recognition [27].

**Figure 8:**
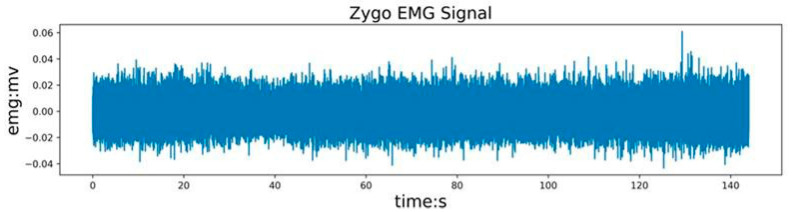
Original sEMG signal with more noise.

**Figure 9:**
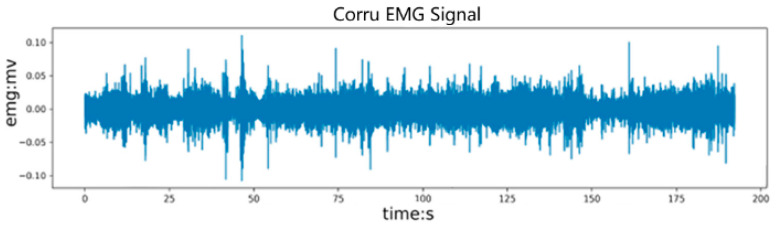
Filtered sEMG signal waveform.

The unprocessed original sEMG signal is shown in Figure 8. A 200 Hz low-pass filter is used to filter out high-frequency interference signals, followed by a 20 Hz low-pass filter to remove interference caused by motion wake. Finally, a 50 Hz notch filter is used to remove power frequency interference. The filtered electromyography signal is shown in Figure 9.

**Figure 10:**
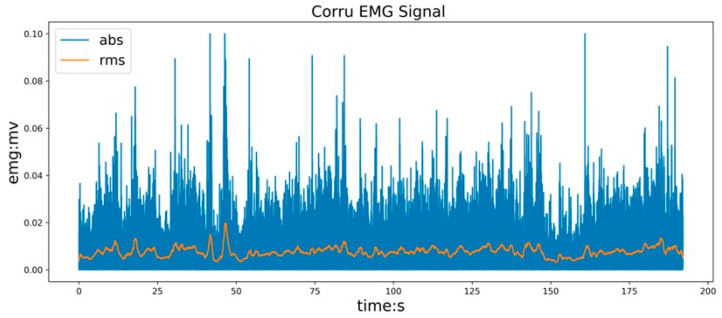
sEMG signal after feature extraction.

The time-domain characteristics are analyzed based on the amplitude of the time series of the sEMG signals, which can effectively reflect muscle strength and frequency. The time-domain features of the MAV and RMS values of the sEMG signals were extracted, as shown in Figure 10.

(1) $$\mathrm{MAV}=\frac{1}{N}\sum_{i=1}^{N}\left|x_{i}\right|,\mathrm{RMS}=\sqrt{\frac{1}{N}\sum_{i=1}^{N}x_{i}^{2}}.$$

where *N* is the total number of single channel samples, and *x*_*i*_ is the numerical value at sampling point *i *.

**Figure 11:**
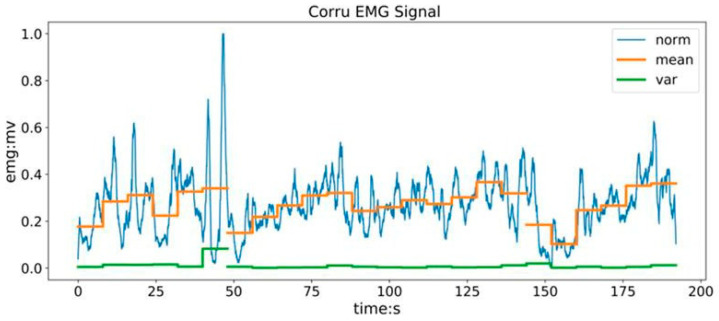
sEMG eigenvalue extraction and normalization processing results.

To eliminate the influence of individual differences caused by multiple acquisitions, it is necessary to normalize the sEMG signals. Select the Interval Scaling Method for normalization, which uses the maximum and minimum feature values to normalize the sEMG signals. After processing, the extracted feature values can be normalized to the (0, 1) range, thus removing differences between the data. The normalized result is shown in Figure 11, and its calculation formula can be expressed as:

(2) $$x^{\prime }=\frac{x_{i}-x_{\min}}{x_{\max}-x_{\min}}.$$

where *x*_*i*_ is the origin feature; *x*_min_ is the minimum eigenvalue; *x*_max_ is the maximum eigenvalue; *x*′ is the new eigenvalue.

### 3.2. Establishment of Hybrid Network Model

**Figure 12:**
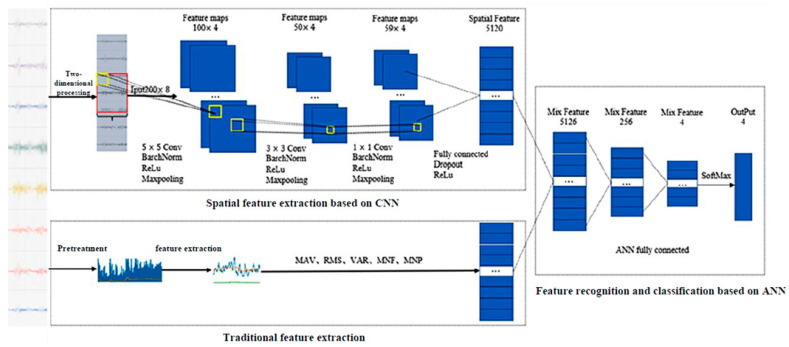
Network structure of the gesture recognition algorithm.

Based on the 8 × 200 matrix (channel × time) obtained from the MYO armband, a ConvNet architecture is implemented using PyTorch 2.0. The non-linear activation function used is the ReLU function. After meticulous optimization to achieve a balanced convergence speed and the capacity to accurately identify an optimal minimum, thereby boosting the training process’s efficiency and effectiveness, the learning rate for the ConvNet [28] is set to 0.00681292, and the dropout rate is set at 0.5, with a batch size of 128. The specific network architecture is shown in Figure 12.

Due to the fact that different channels of sEMG data correspond to different positions on the arm, convolution and pooling operations on the sEMG images are performed only in the length direction of the image, which allows extraction of temporal features from the signals and ensures the independence of data between different channels. After performing the three convolution and two pooling operations on the original data, the resulting data has dimensions of (64, 128, 5, 8). Flattening the data into vector form yields data with dimensions of (64, 5120).

On the other hand, since the characteristics of sEMG signals are also contained in the frequency domain and time-frequency domain, extracting frequency domain features after the Fourier transform of the original sEMG signal. Since the RMS value of the amplitude of the electromyography signal can best reflect the degree of muscle activity corresponding to the electromyography signal, the authors also manually extracted the RMS feature value of the sEMG signal. Then, the manually extracted feature vectors and the feature vectors obtained by the convolutional neural network are concatenated as inputs to the fully connected network. After calculation by two layers of fully connected networks, the score vectors for each gesture category are finally obtained.

### 3.3. Analysis of Gesture Recognition Results

**Figure 13:**
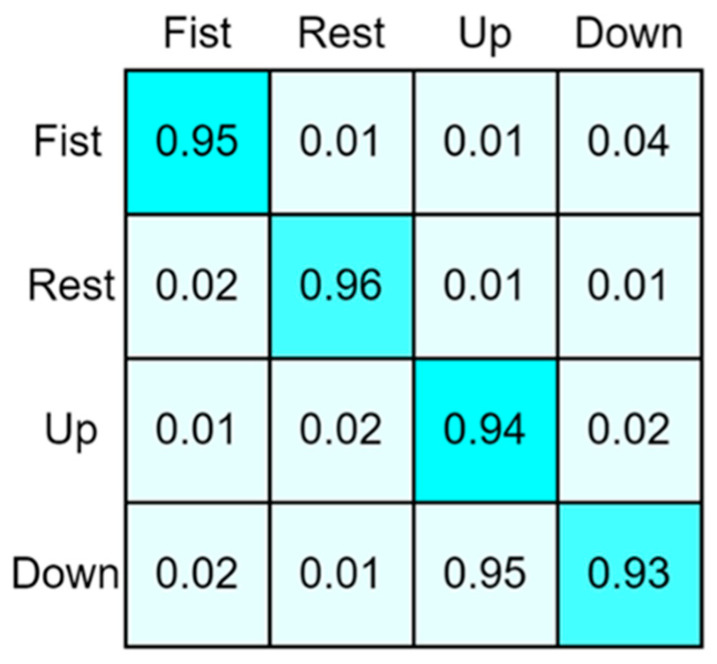
Gesture recognition results.

The confusion matrix being trained by the CNN–ANN Hybrid Network Model [29,30,31] with multi-feature fusion is shown in Figure 13.

According to the confusion matrix, it is evident that clenching and relaxing movements are relatively easy to recognize, and the recognition rates of both movements are above 95%. Although the accuracy of distinguishing between palm-up and palm-down movements is not high, the recognition accuracy can still reach over 90%, and the overall average recognition rate is 94.50%. Actual analysis also finds that there are obvious changes in the muscle movements used during clenching and relaxation states, and the muscle groups that control movement are not closely connected. Conversely, the muscles involved in palm-up and palm-down flipping exhibit closer connections, which potentially cause interference in sEMG signal collection. Therefore, when designing palm gestures for sEMG, selecting gestures with significant differentiation in muscle activity positions and ample spacing between muscle activities can improve the performance of sEMG gesture recognition systems.

## 4. Arm Action Recognition

### 4.1. Establishment of BP Neural Network Model

The focus of the arm movement prediction model is to establish a regression model that takes input from electromyography signals and arm joint movement angles to predict the various arm movement angles. In supervised learning utilizing BP Neural Network, the initial phase encompasses the forward propagation of input data, progressing from the input layer to the output layer in a sequential manner. Following this, the discrepancy between the actual output results and the arm joint movement angles is backpropagated from the output layer all the way to the input layer. Adjusting the weights and the thresholds in the network parameters to a certain extent allows the error function (E) to decrease in a negative gradient mode, thereby achieving optimal results [32].

**Figure 14:**
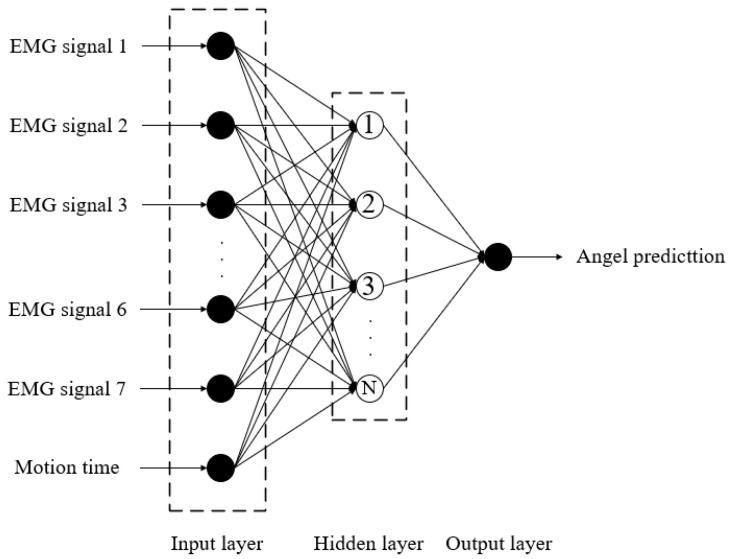
The neural network structure depicted in the figure is based on the research conducted in this paper.

A three-layer BP Neural Network algorithm is selected to establish an angle prediction model, as depicted in Figure 14. The input layer, serving as the first layer of the network, utilizes a total of eight nodes, signifying that there are eight input variables. These variables represent the sEMG signals from 1 to 7 (corresponding to the signals from seven muscles) and the motion time. Subsequently, the middle layer comprises one hidden layer, for which the number of nodes, determined to be 20 through a trial method, is established through continuous network training. The final layer is the output layer, representing the estimation of joint motion angle data.

In practical applications, the BP algorithm suffers from slow convergence and the presence of local minima in the objective function, therefore necessitating some modifications to the BP algorithm [33,34].

1. Incorporate momentum term into the BP Neural Network.

The addition of a momentum term is aimed at the backpropagation process, where a quantity proportional to the previous weight change is added to each weight adjustment, thereby generating a new weight adjustment. Its mathematical expression can be defined as follows:

(3) $$\Delta w_{y}(k+1)=(1-mc)\eta \delta_{i}p_{j}+mc\Delta w_{y}(k),$$

(4) $$\Delta b_{i}(k+1)=(1-mc)\eta \delta_{i}+mc\Delta b_{i}(k).$$

where *k* is the number of training iterations, and *mc* is the momentum factor.

By adding momentum terms during network training, small adjustments can be made to the weight correction while also avoiding entering local minima during the learning process.

2. Modify adaptive learning rate.

The criterion for adaptive adjustment of the learning rate is to compare the change in the error function from each iteration. If the error function decreases compared to the previous iteration, then increase the learning rate. If the error function does not decrease and the adjustment is too large, then reduce the learning rate. Its mathematical expression is as follows:

(5) $$\left\{\begin{array}{cc} 1.05\cdot \eta (k) & SSE(k+1)<SSE(k)\\ \;0.7\cdot \eta (k) & SSE(k+1)>1.04SSE(k)\\ \;\;\;\;\eta (k) & others \end{array}\right..$$

where *SSE* is the sum of squared errors.

### 4.2. Analysis of Joint Single-Degree-of-Freedom Test Results

**Figure 15:**
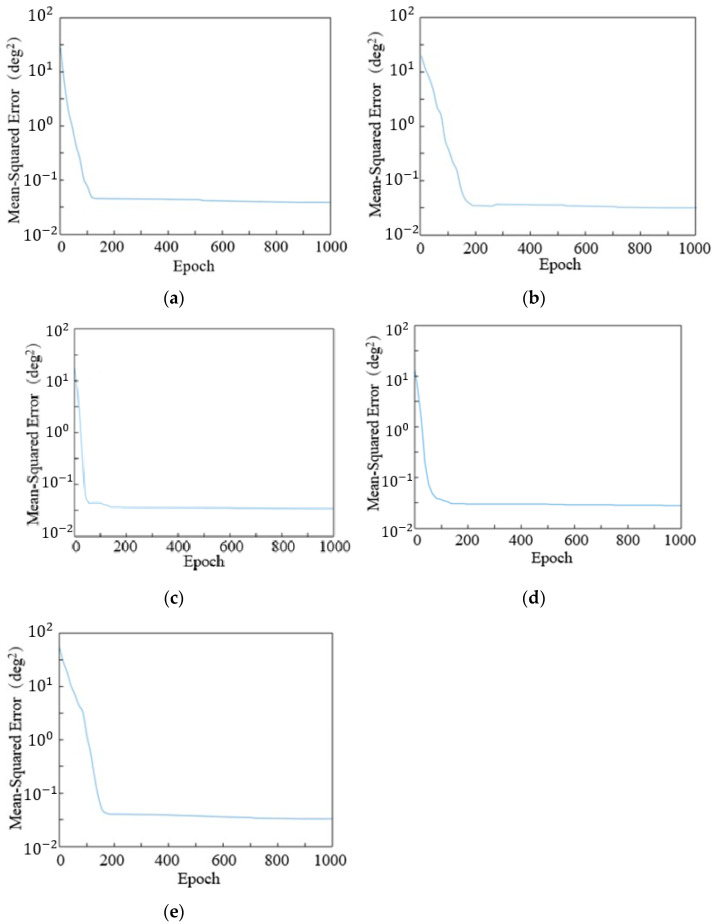
Error curve of each joint movement: (**a**) Shoulder abduction/adduction. (**b**) Shoulder flexion/extension. (**c**) Elbow flexion/extension. (**d**) Forearm rotation. (**e**) Shoulder rotation.

The collected sEMG signal dataset from each joint’s single-degree-of-freedom motion is used to train the neural network. Figure 15 shows the error curve of the network training. Mean-squared error (MSE) represents the sum of squares of errors between the actual output values and the target output values:

(6) $$\mathrm{MSE}=\frac{1}{n}\sum_{i=1}^{n}w_{i}{\left(Y_{i}-{\hat{Y}}_{i}\right)}^{2}.$$

where *n* is the number of data; *Y*_*i*_ is the actual value; ${\hat{Y}}_{i}$ is the predicted value.

**Figure 16:**
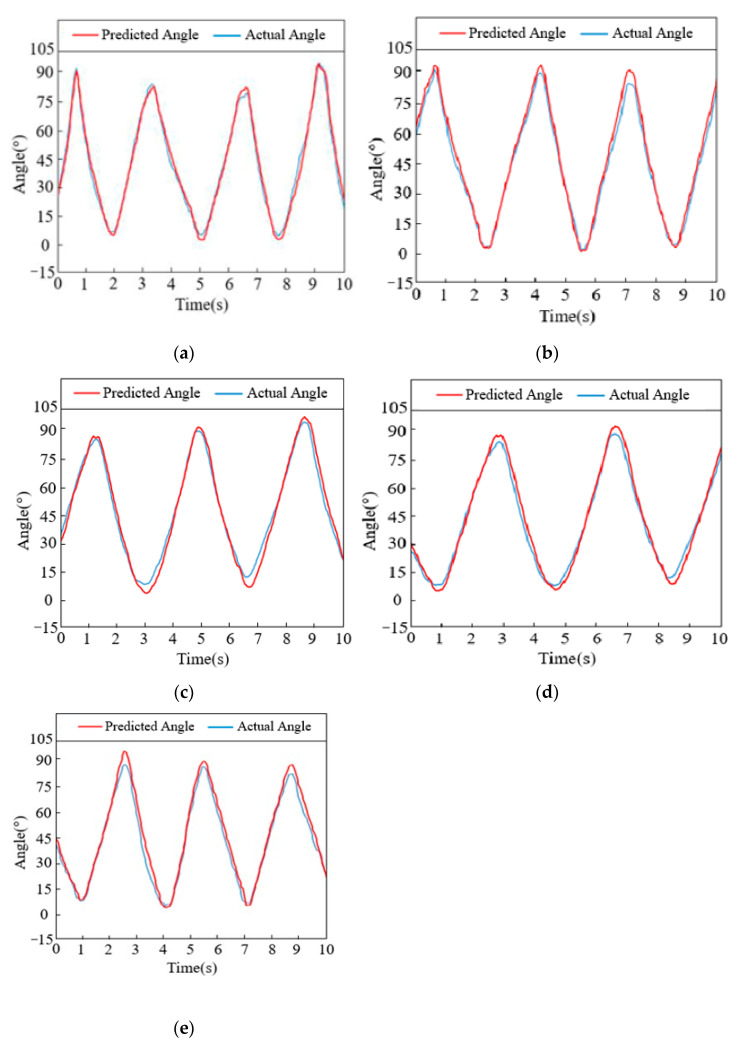
Comparison of target and predicted angles during joint movement: (**a**) Shoulder abduction/adduction. (**b**) Shoulder flexion/extension. (**c**) Elbow flexion/extension. (**d**) Forearm rotation. (**e**) Shoulder rotation.

Then, input the sEMG signal data from each action test set into the trained neural network for joint angle prediction. The target angle and predicted angle curves obtained are shown in Figure 16.

As can be seen from the figure, the trends of the two curves in the figure are almost identical. The larger errors occur at the initial moment of the single-joint motion range, which is mainly due to the time difference in the normalization processing of the electromyographic signals. Therefore, based on the constructed neural network model, experiments on the estimation of joint angles during continuous motion can be conducted.

### 4.3. Analysis of Continuous Motions Test Results

**Figure 17:**
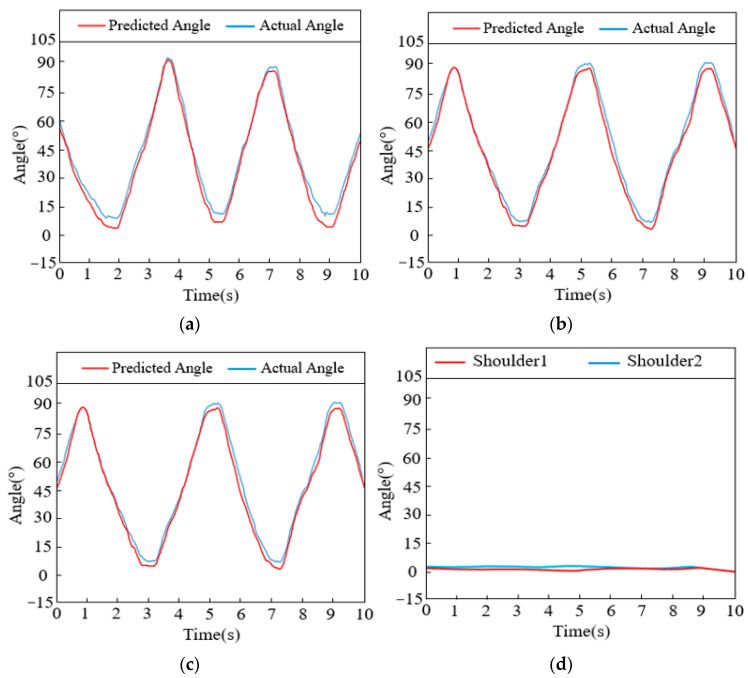
Intersection curve of each joint (the combined action of abduction and raising of the arm): (**a**) Shoulder abduction/adduction. (**b**) Elbow flexion/extension. (**c**) Forearm rotation. (**d**) Shoulder flexion/extension and rotation.

For the combined action of abduction and raising of the arm, the gesture recognition results obtained after processing the collected data are shown in Table 2, with the predictions of joint angles illustrated in Figure 17. Figure 17a–c depict the actual and predicted angle curves for shoulder abduction/adduction, elbow flexion/extension, and forearm rotation, respectively. Meanwhile, during the motion, the shoulder joint did not undergo flexion/extension or rotation movements. Figure 17d shows the angle prediction curves for these two degrees of freedom of the shoulder joint where no action occurred.

**Figure 18:**
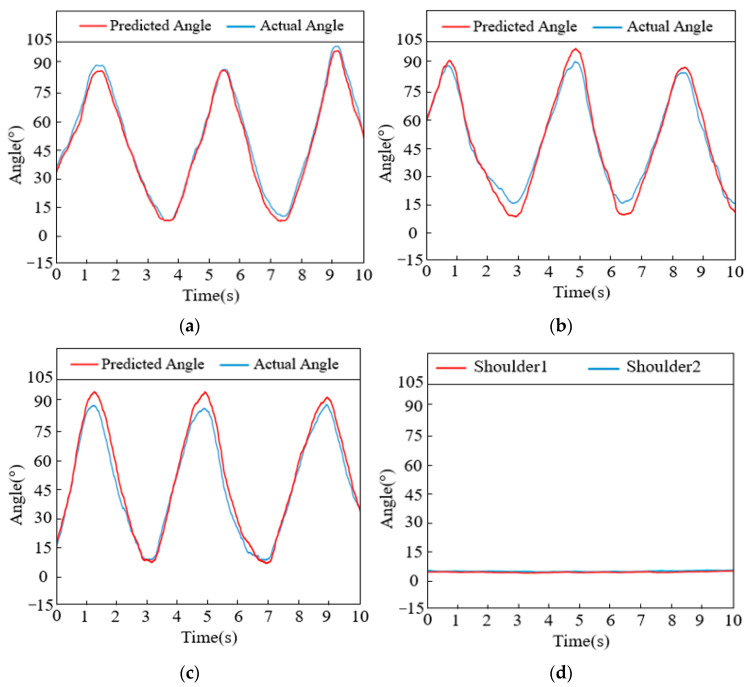
Intersection curve of each joint (the combined action of the cross arm with palm clenched): (**a**) Shoulder abduction/adduction. (**b**) Elbow flexion/extension. (**c**) Forearm rotation. (**d**) Shoulder flexion/extension and rotation.

For the combined action of the cross arm with palm clenched, the gesture recognition results obtained after processing the collected data are shown in Table 3, with the predictions of joint angles presented in Figure 18. Figure 18a–c, respectively, display the actual and predicted angle curves for shoulder flexion/extension, elbow flexion/extension, and forearm rotation. Figure 18d shows the predicted angle curves for the two unused degrees of freedom during this action: shoulder abduction/adduction and rotation.

Although the difficulty of recognizing multiple actions performed simultaneously is greater than that of single-joint movements, based on the experimental data above, it can be determined that when movements involving three degrees of freedom occur simultaneously in various joints of the arm, the predicted joint angles tend to have a slightly larger error compared to single-joint movements. However, the overall trend of the target and predicted angle curves is similar, and the curves exhibit a high degree of fit. The maximum error does not exceed 5°. Despite a certain impact on the recognition rate, it does not prevent the action from being recognized. 

Also, degrees of freedom that do not undergo movement can be accurately identified. Figure 17d and Figure 18d clearly demonstrate that the predicted angles consistently remain between 1° and 2°, which can be determined as no action occurring in those degrees of freedom. Compared to joint angle prediction, gesture action recognition does not decrease in accuracy due to multiple actions being performed simultaneously, and it still maintains a high recognition rate. Compared to existing research, the human motion mapping method established in this paper based on sEMG signals can better predict the joint angles of continuous movements and provides a new research approach for predicting the joint angles of combined movements.

## 5. Human–Machine Action Mapping Experiment

**Figure 19:**
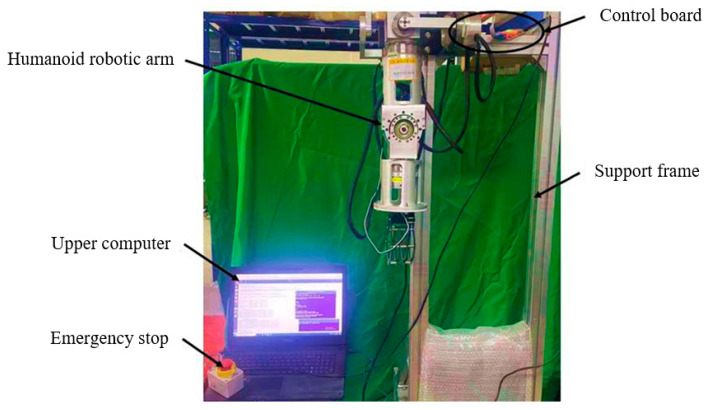
Physical drawing of the humanoid manipulator.

**Figure 20:**
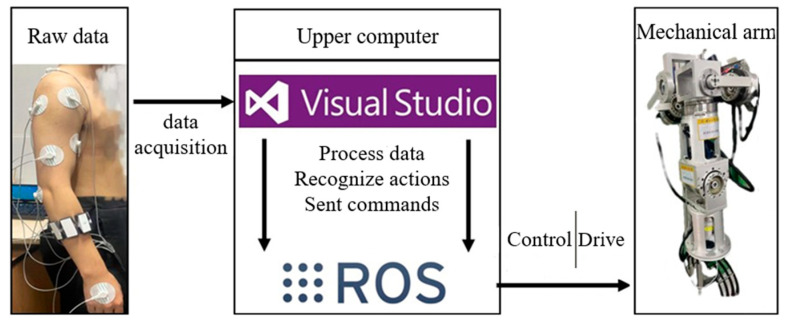
Schematic diagram of test system.

The experimental platform constructed [22,23,24] is shown in Figure 19, and the designed test system is illustrated in Figure 20. An operator wears an sEMG signal acquisition device and performs actions, which are captured as raw EMG signals and transmitted to the upper computer software in Visual Studio. Through a constructed action recognition program, the raw sEMG signals undergo a series of data processing and recognition. Ultimately, the recognized human limb movements are converted into control commands. These commands are then sent to the control system of the robotic arm. Upon receiving the commands, the control system transmits motion parameters to the robotic arm, which then completes the corresponding limb movements.

The task process is divided into the following four parts: 

Step A: Control the elbow and shoulder joints to bend forward, allowing the robotic claw to reach the position of the water bottle. 

Step B: Command the robotic claw to close and grasp the water bottle. 

Step C: Control the forearm to rotate, tilting the water bottle so water pours out from its mouth. 

Step D: Control the forearm to rotate back to its initial position and command the robotic claw to release the water cup. Finally, control the elbow and shoulder joints to return to their initial positions.

**Figure 21:**
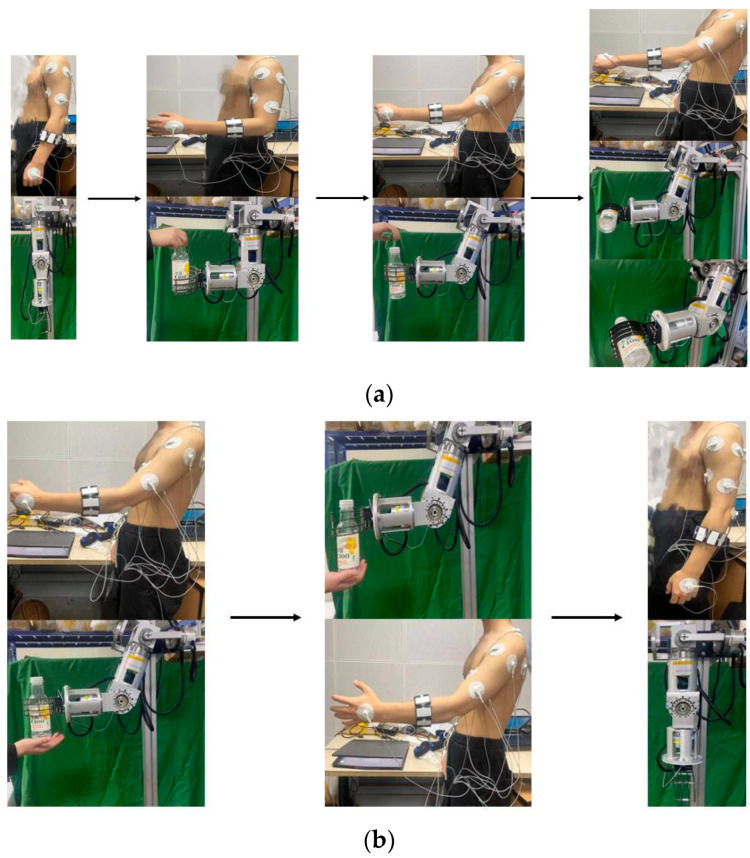
Experiment on control of humanoid robot arm: (**a**) Perform water pouring action. (**b**) Restore initial state action.

These experimental actions are carried out a total of 20 times, and the success rate is determined by the number of completions. The experimental process is shown in Figure 21, and the experimental results are presented in Table 4.

The overall motion process consists of two parts: performing the water-pouring task and returning to the initial state. The critical actions within this process are grasping the target water bottle and rotating the arm to achieve the goal of tilting the bottle to pour water. The entire experiment proceeded smoothly, successfully completing an experiment in human–machine interaction that involved recognizing human movements through sEMG signals and mapping those movements onto a humanoid robotic arm.

## 6. Conclusions

This paper explores the recognition of arm movements, including shoulder abduction and adduction, flexion and extension, rotation, elbow flexion and extension, forearm rotation, and four hand gestures, through an sEMG signal-joint angle model. The study establishes a mapping model between sEMG signals and movement angles by extracting features and employing a backpropagation neural network, resulting in high-accuracy predictions of joint single-degree-of-freedom movements and combined movements’ angles. Additionally, our work accomplished the complete action of pouring water by mapping actions to a robotic arm using sEMG signals, demonstrating the feasibility of applying sEMG signals to robotic arm control. The results indicate that the sEMG signal-joint angle mapping model is an efficient motion recognition algorithm and plays an important role in human–computer interaction. However, it must be noted that human limb activities are diverse, and muscular conditions vary, presenting numerous problems and challenges that need to be addressed in practice. Furthermore, with the development of deep learning models, considering the use of transformer-based pre-trained models through practical transfer learning algorithms to reduce training iterations is a research direction worth exploring.

## Figures and Tables

**Table 1:** Joint motion planning.

Joints	Motion	Range (°)
Shoulder	Abduction/Adduction	0–90
	Flexion/Extension	0–90
	Internal rotation/External rotation	0–90
Elbow	Flexion/Extension	0–120
	Internal rotation/External rotation	−30–150

**Table 2:** Gesture recognition rate of the combined action of abduction and raising of the arm.

Motion	Successful Recognition	Failed Recognition	Succes s Rate
Palm relax	48	2	96%

**Table 3:** Gesture recognition rate of the combined action of the cross arm with palm clenched.

Motion	Successful Recognition	Failed Recognition	Success Rate
Palm clench	49	1	98%

**Table 4:** Manipulator control success rate.

Number	1	2	3	4	5	Success Rate
A	4/4	4/4	3/4	4/4	4/4	95%
B	4/4	4/4	4/4	4/4	4/4	100%
C	4/4	4/4	4/4	4/4	4/4	100%
D	4/4	3/4	4/4	4/4	2/4	85%

## Data Availability

Data are contained within the article.
